# Prognostic value of LDH and α-HBDH dynamic levels for 1-year overall survival and progression-free survival in patients with extensive-stage small-cell lung cancer treated with chemoimmunotherapy

**DOI:** 10.3389/fonc.2025.1721581

**Published:** 2026-01-12

**Authors:** Wanjing Li, Guochang Du, Shuangqing Lu, Hui Zhu, Ke Zhao, Jinming Yu, Yanqin Yang

**Affiliations:** 1 Department of Radiation Oncology, Shandong University Cancer Center, Cheeloo College of Medicine, Shandong University, Jinan, Shandong, China; 2 Department of Radiation Oncology, Shandong Cancer Hospital and Institute, Shandong First Medical University and Shandong Academy of Medical Sciences, Jinan, China

**Keywords:** chemoimmunotherapy, extensive-stage small cell lung cancer, lactate dehydrogenase, prognostic biomarkers, α-hydroxybutyrate dehydrogenase

## Abstract

**Background:**

Extensive-stage small cell lung cancer (ES-SCLC) remains a lethal malignancy with limited prognostic biomarkers. This study evaluated the prognostic utility of lactate dehydrogenase (LDH) and α-hydroxybutyrate dehydrogenase (α-HBDH) levels at baseline and after 2 cycles of treatment in ES-SCLC patients receiving first-line chemoimmunotherapy.

**Methods:**

Continuous variables were converted into categorical ones based on optimal cutoffs identified by ROC curve analysis (maximizing Youden’s index). Overall survival (OS) and progression-free survival (PFS) were calculated via the Kaplan–Meier method and compared via the log-rank test. In addition, the Cox regression model was used to analyze prognostic factors. Associations between LDH and α-HBDH levels and clinical parameters (T/N stage, age, and immune-related toxicity) were analyzed using the Mann–Whitney U test and binary logistic regression.

**Results:**

A cohort of 201 ES-SCLC patients treated with chemotherapy and immune checkpoint inhibitors was analyzed retrospectively. Elevated baseline serum LDH >245 U/L and α-HBDH >182 U/L levels predicted poor OS (p = 0.046, HR = 1.798, 95% CI: 1.020–3.167; p = 0.007, HR = 2.268, 95% CI: 1.288–3.994); inadequate 2-cycle reductions (ΔLDH ≤ 108.5U/L; Δα-HBDH ≤ 62.5U/L) further predicted poor OS (p<0.001, HR = 2.561, 95% CI: 1.291–5.080; p<0.001, HR = 2.807, 95% CI: 1.457–5.411). For progression-free survival, no significant difference was observed between patients with elevated baseline serum LDH >245 U/L or α-HBDH >182 U/L and those with normal levels; similarly, there was no significant difference between patients with an on-treatment increase (ΔLDH>12.5 U/L or Δα-HBDH>0.5 U/L from baseline) and those with a lesser increase or decrease. (P = 0.768, HR = 1.055, 95% CI: 0.739–1.507 for baseline LDH; P = 0.529, HR = 1.121, 95% CI: 0.785–1.601 for baseline α-HBDH; P = 0.115, HR = 0.719, 95% CI: 0.457–1.131 for ΔLDH; P = 0.094, HR = 0.730, 95% CI: 0.494–1.080 for Δα-HBDH). Advanced T4/N3 stage and age ≥65 years significantly modulated biomarker trajectories (p<0.05).

**Conclusion:**

LDH and α-HBDH serve as key prognostic biomarkers in ES-SCLC patients undergoing first-line chemoimmunotherapy. We established clinically validated thresholds, which can guide treatment by identifying patients with elevated or insufficiently declining levels as high-risk, thereby supporting early and targeted therapeutic intervention.

## 1 Introduction

Small cell lung cancer (SCLC), accounting for 20–25% of all lung cancer cases, is highly aggressive with rapid growth and early metastasis. Approximately 70% of patients present with extensive-stage SCLC (ES-SCLC) at diagnosis (1, 2). Although ES-SCLC is initially sensitive to chemotherapy and radiation therapy, the prognosis remains dismal. Patients with ES-SCLC have a poor prognosis, with a median OS of 7.5–10.9 months and a 5-year OS rate of only 2.8% (3). The advent of immune checkpoint inhibitors has greatly advanced the treatment of ES-SCLC. The integration of immunotherapy with chemotherapy, established by pivotal trials (IMpower133, CASPIAN, CAPSTONE-1), has become the first-line standard, modestly extending survival (4–7).

ES-SCLC is characterized by rapid proliferation and metabolic reprogramming (8). Tumor cells preferentially utilize glycolysis over oxidative phosphorylation even in oxygen-sufficient conditions (the Warburg effect) to fuel growth (9, 10). In this context, lactate dehydrogenase (LDH) and its isoenzyme α-hydroxybutyrate dehydrogenase (α-HBDH) serve as key enzymes that orchestrate the Warburg effect. LDH maintains glycolytic flux by catalyzing the conversion of pyruvate to lactate (11). As an LDH isoenzyme, α-HBDH sustains aerobic glycolysis by catalyzing pyruvate-to-lactate conversion and NAD^+^ regeneration, thereby preserving glycolytic flux in cancer cells. Functional studies have further established the critical role of LDH: genetic knockout or pharmacological inhibition of LDH expression significantly reduces lactate production and suppresses glycolytic flux in cancer models (12). Consequently, elevated levels of LDH and α-HBDH indicate enhanced Warburg effect metabolism in tumors. Concurrently, lactate overproduction induced by this process acidifies the tumor microenvironment (TME). This acidosis not only promotes tumor invasion but also suppresses antitumor immunity by inhibiting cytotoxic T cells and polarizing macrophages toward a protumor phenotype via mechanisms such as pH-sensitive PD-L1 upregulation (13, 14). Consequently, persistently elevated LDH and α-HBDH levels are directly correlated with tumor progression and poor immunotherapy outcomes.

Current research on LDH and α-HBDH has focused primarily on hematologic malignancies and ovarian cancer. Studies have confirmed that serum levels of these enzymes are significantly elevated in patients with diverse cancers (e.g., lymphoma, ovarian cancer, and lung cancer) (15–17). However, the association between LDH/α-HBDH dynamics and the efficacy of immunotherapy remains underexplored. Moreover, studies have rarely established thresholds with sufficient prognostic sensitivity for early monitoring. To address these gaps, we developed validated dynamic thresholds that enhance specificity and uniquely interrogate immunometabolic crosstalk in the ES-SCLC microenvironment. Therefore, in this retrospective cohort study, the prognostic value of longitudinally monitored serum LDH/α-HBDH levels and their dynamic changes during first-line chemoimmunotherapy was evaluated in ES-SCLC patients. It is also hoped that validated serum LDH and α-HBDH thresholds can be established to guide the treatment of ES-SCLC patients.

## 2 Materials and methods

### 2.1 Study population

**Figure 1:**
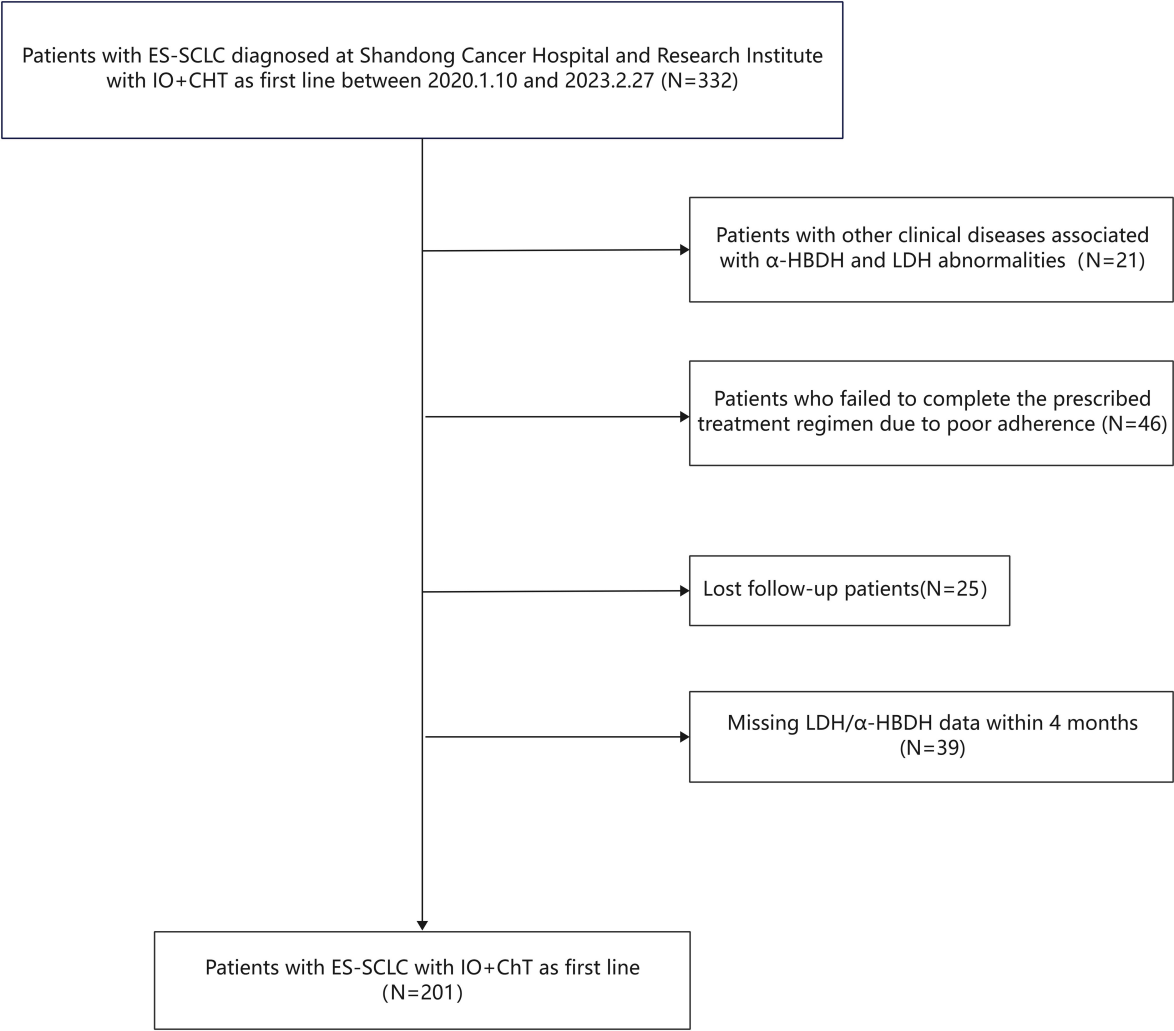
Flowchart of the screening procedure.

Serum LDH and α-HBDH levels were collected from patients with ES-SCLC who were hospitalized at Shandong Cancer Hospital between January 10, 2020, and February 27, 2023, at baseline and after 2 cycles of first-line chemoimmunotherapy. Patients with other clinical conditions known to affect LDH and α-HBDH levels—including cardiac diseases, muscular dystrophy, vitamin B12 deficiency, hemolytic anemia, renal infarction, renal vascular embolism, and cachexia—were excluded. In addition, patients who missed 2 consecutive treatment cycles were excluded. The patient screening flowchart is illustrated in **Figure 1**.

A total of 201 eligible ES-SCLC patients—162 men and 39 women—with a median age of 61 years (range: 39–82 years) were ultimately included. Clinicopathologic data were obtained from electronic medical records and included demographic information; smoking history; tumor stage; metastasis site (liver, kidney, brain, or bone); laboratory values (routine, biochemical, and tumor markers); type of immunotherapy used; presence of pretreatment pulmonary conditions (e.g., obstructive pneumonitis and interstitial lung disease); and posttreatment immune-related adverse events (irAEs), defined according to ASCO guidelines. These irAEs included immune-mediated myocarditis (elevated troponin-I and cardiac MRI-confirmed inflammation), hypothyroidism (TSH >4.0 mIU/L with relevant symptoms), and pneumonia (a new infiltrative shadow confirmed by chest CT with typical infection excluded as the cause).

### 2.2 Follow−up

Patient data were retrieved from the electronic medical records system of Shandong Cancer Hospital and screened according to the defined inclusion and exclusion criteria. Follow-up data were collected via telephone interviews and a review of follow-up records, and survival status, time and cause of death, and current health condition were documented. The cutoff date for follow-up was June 14, 2024. Follow-up data were used to calculate one-year OS and PFS. Cases involving death unrelated to tumor progression—such as accidental or unrelated disease-related deaths—were excluded from the analysis.

### 2.3 Statistical methods

In this study, receiver operating characteristic (ROC) curves were first generated to determine the optimal cutoff values for noncategorical variables. After sensitivity and specificity were considered, critical values were determined on the basis of the maximum Youden’s index. Continuous variables were then converted to categorical variables. Kaplan–Meier survival curves were generated to assess OS and PFS, with between-group differences evaluated using the log-rank test. Univariate and multivariate Cox proportional hazards models were used to identify potential prognostic factors. Associations between LDH and α-HBDH levels and clinical parameters were analyzed using the Mann–Whitney U test and binary logistic regression. All statistical analyses were performed using SPSS version 23.0, with a p value of < 0.05 considered to indicate statistical significance. Kaplan–Meier survival curves were generated using R v4.4.2 (survival package).

## 3 Results

### 3.1 Basic information and characteristics

A total of 201 patients with ES-SCLC were included in the analysis. First-line treatment for all patients consisted of chemotherapy combined with immunotherapy. The patient characteristics are shown in **Table 1**. PD-1 inhibitors were administered to 85 patients (42.3%), whereas PD-L1 inhibitors were given to 116 patients (57.7%). The cohort included 162 men and 39 women, with a median age of 61 years. A history of smoking was reported for 119 patients (59.2%). The main metastatic sites included the brain (29.4%, n=59), bone (23.4%, n=47), liver (32.8%, n=66), adrenal glands (15.4%, n=31). Pretreatment pneumonia (obstructive, interstitial, or infectious) was present in 78 patients (38.8%). IrAEs following treatment included immune-related pneumonitis (13.9%, n=28), myocarditis (13.9%, n=28), and hypothyroidism (17.4%, n=35).

**Table 1:** Baseline patient characteristics.

Characteristic	Total (%) (n=201)
Sex	
Male	162(80.6)
Female	39(19.4)
Age(years)	
(Median, range)	61(39-82)
<65	134(66.7)
>65	67(33.3)
Smoking status	
Yes	119(59.2)
No	82(40.8)
T stage	
1-3	119(59.2)
4	82(40.8)
N stage	
0-2	105(52.2)
3	96(47.8)
Baseline metastasis site	
Brain	59(29.4)
Bone	47(23.4)
Liver	66(32.8)
Adrenal gland	31(15.4)
Pneumonia (obstructive	
pneumonia, interstitial	
pneumonia, infections)	
Yes	78(38.8)
No	123(61.2)
Immunogenic pneumonia	
Yes	28(13.9)
No	173(86.1)
Immune mediated myocarditis	
Yes	28(13.9)
No	173(86.1)
Immunologic thyroid abnormalities	
Yes	35(17.4)
No	166(82.6)
Immune checkpoint inhibitors	
PD-1 inhibitors	85(42.3)
PD-L1 inhibitors	116(57.7)

### 3.2 Survival outcomes analysis

**Figure 2:**
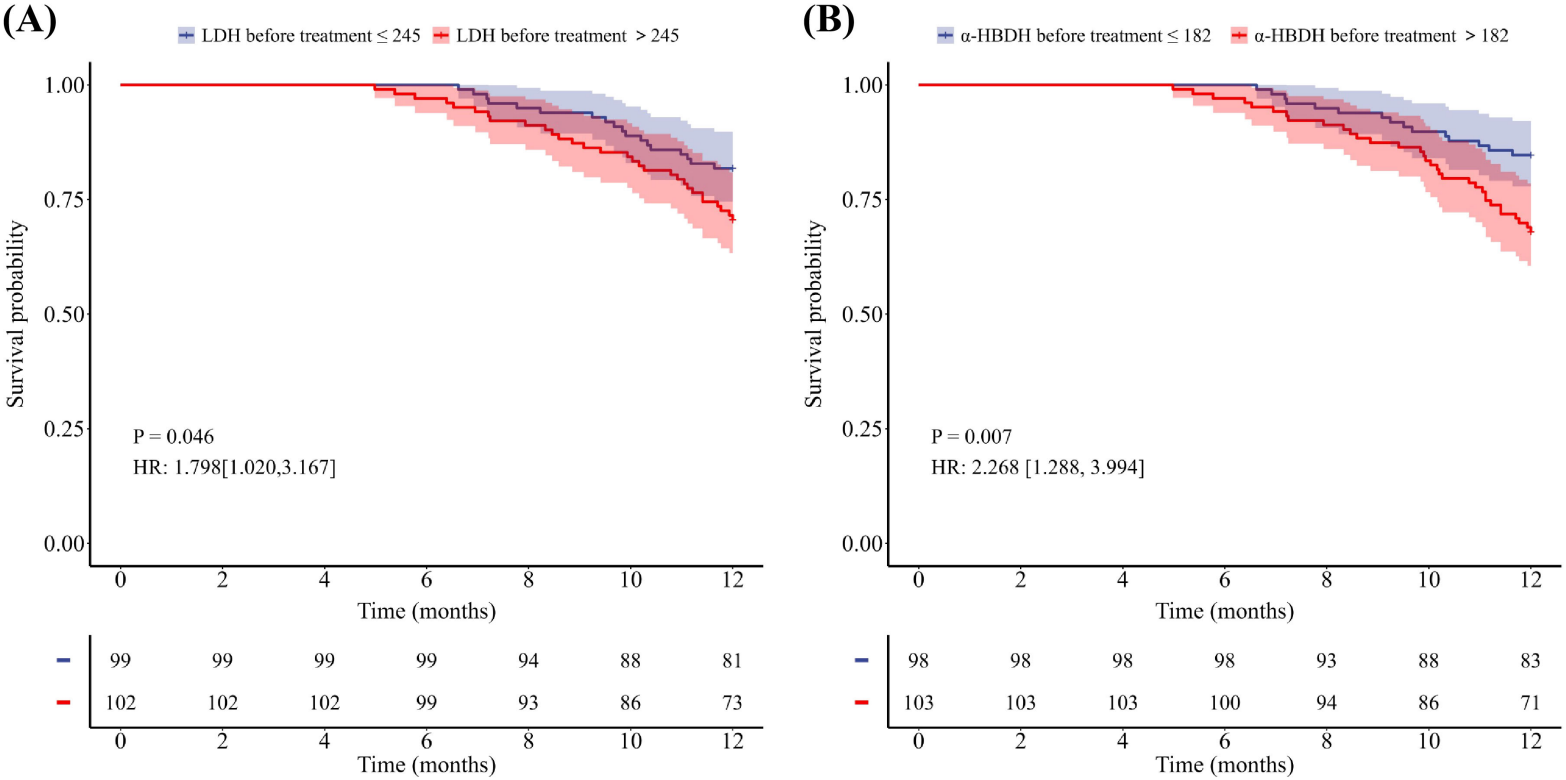
Kaplan-Meier curves for OS stratified by baseline serum levels. **(A)** OS according to LDH levels (>245 U/L vs ≤245 U/L). **(B)** OS according to a-HBDH levels (>182 U/L vs ≤182 U/L).

**Figure 3:**
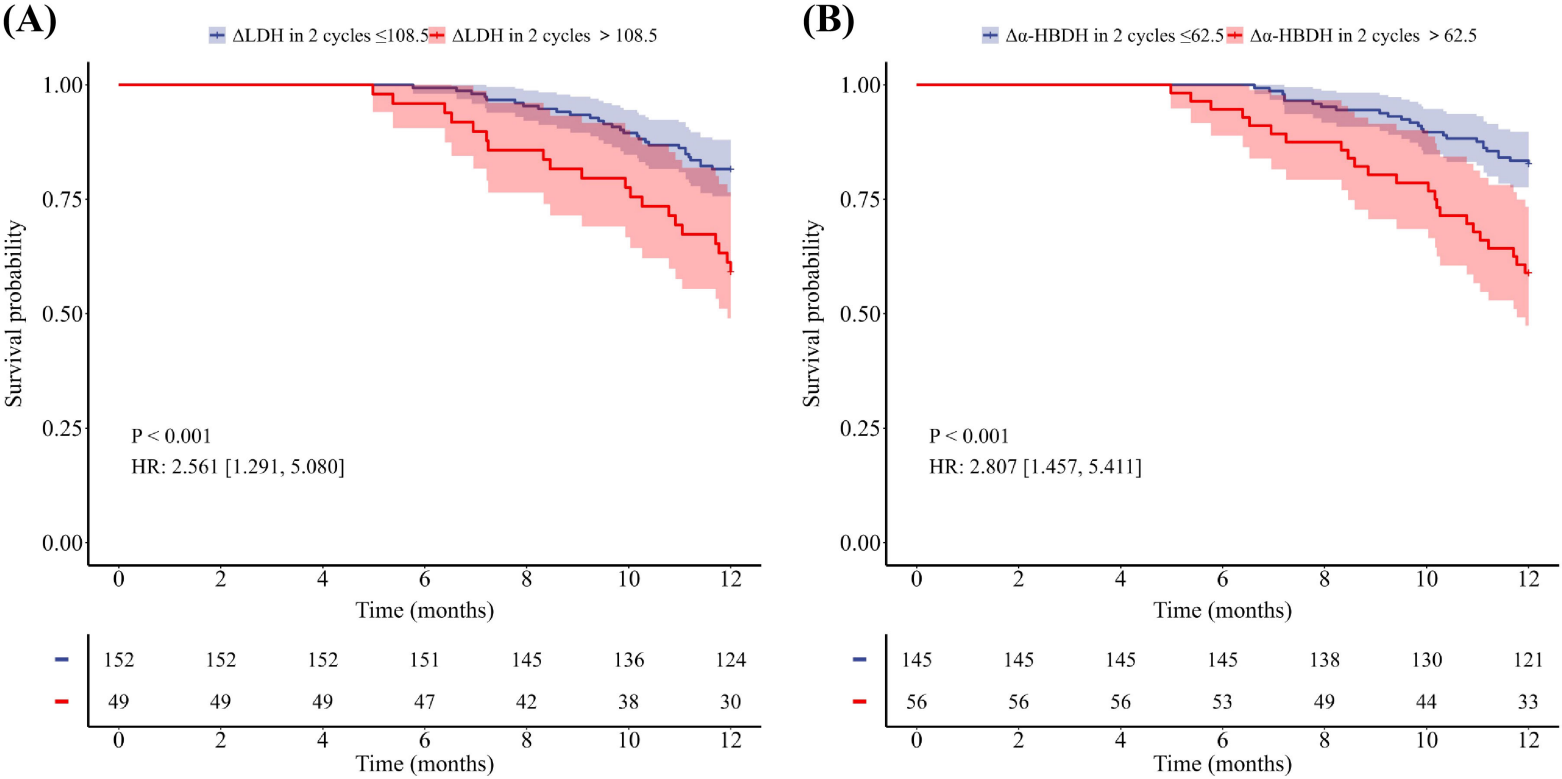
Kaplan-Meier curves for OS stratified by the reduction after two treatment cycles. **(A)** OS according to the magnitude of LDH reduction (ΔLDH ≤ 108.5 U/L vs >108.5 U/L). **(B)** OS according to the magnitude of a-HBDH reduction (Δa-HBDH ≤ 62.5 U/L vs >62.5 U/L).

**Figure 4:**
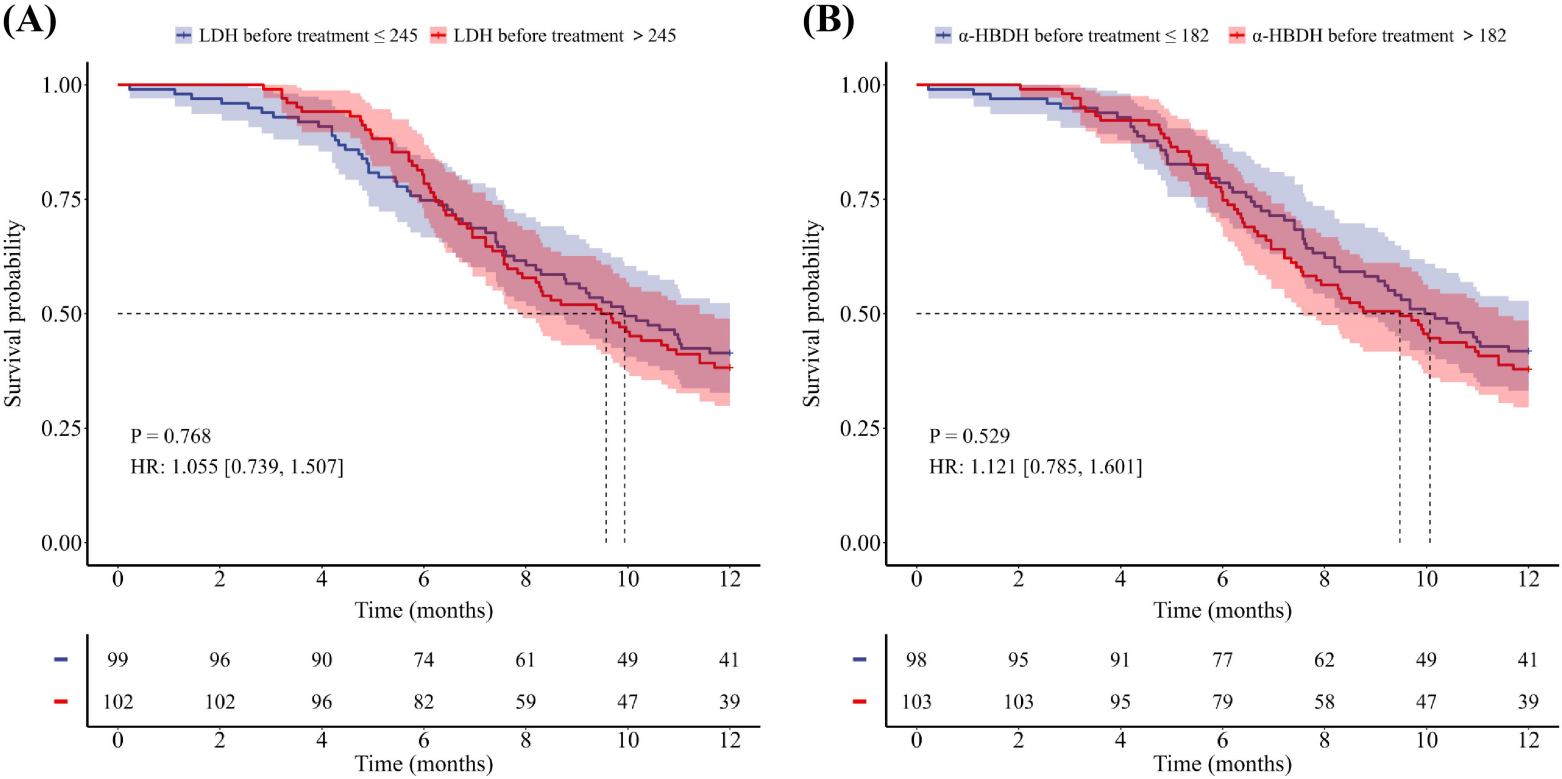
Kaplan-Meier curves for PFS stratified by baseline serum levels. **(A)** PFS according to LDH levels (>245 U/L vs ≤245 U/L). **(B)** PFS according to a-HBDH levels (>182 U/L vs ≤182 U/L).

**Figure 5:**
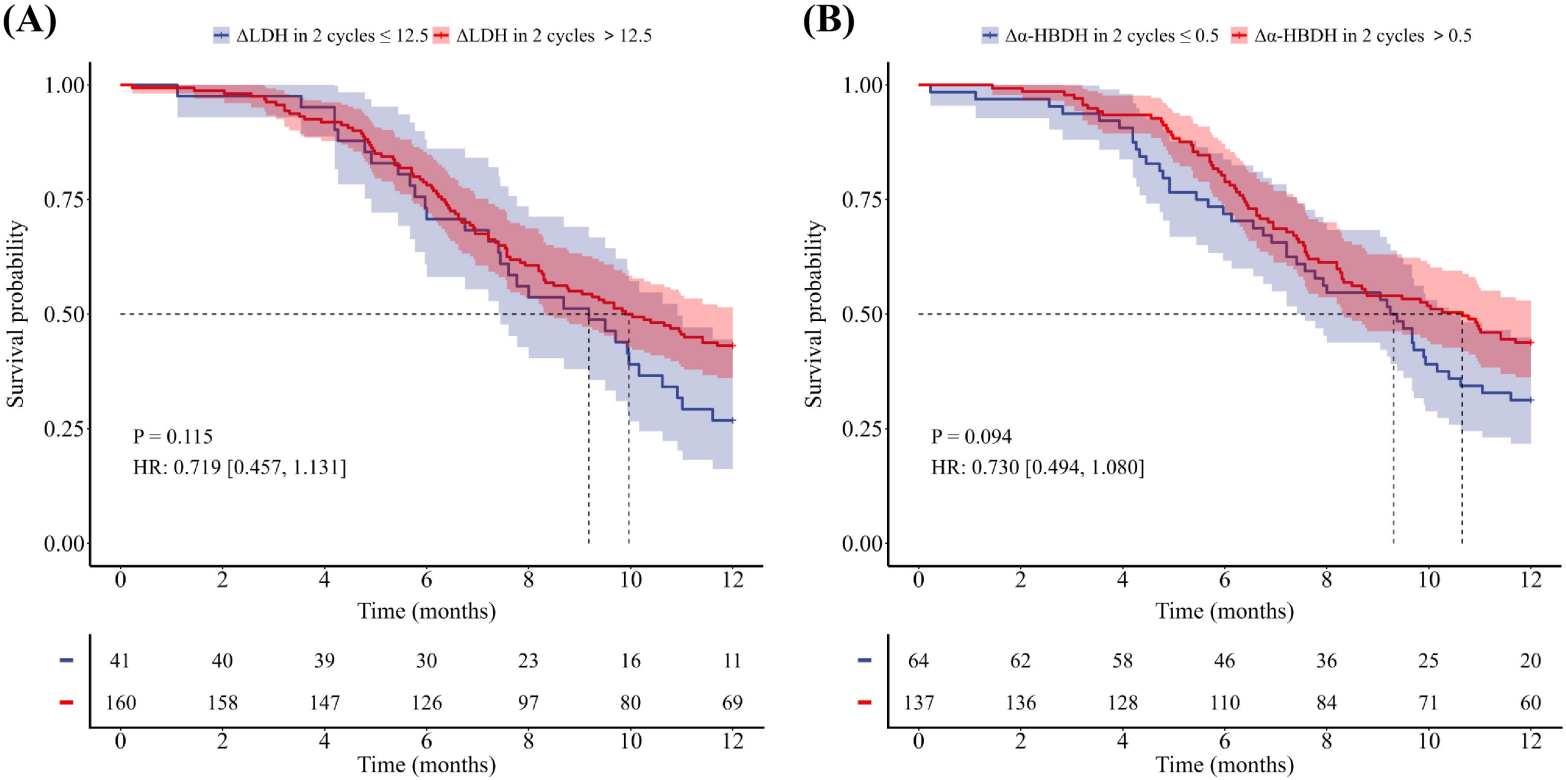
Kaplan-Meier curves for PFS stratified by on-treatment changes. **(A)** PFS according to LDH increase (ΔLDH >12.5 U/L vs ≤12.5 U/L). **(B)** PFS according to a-HBDH increase (Δa-HBDH >0.5 U/L vs ≤0.5 U/L).

Of the 201 patients, 48 patients died within one year, 122 patients experienced disease progression within one year, and the median PFS for the entire population was 9.7 months, with a median OS not reached. As shown in **Tables 2** and **3**, univariate Cox analyses revealed that sex, age, ΔLDH after two treatment cycles, baseline α-HBDH, and Δα-HBDH after two treatment cycles were significant predictors of 1-year overall survival (all *P* < 0.05), whereas baseline bone metastasis emerged as a key predictor of 1-year progression-free survival (*P* < 0.05). Multivariate analysis confirmed that sex, age, and Δα-HBDH after two cycles independently predicted 1-year overall survival (*P* < 0.05), whereas baseline bone metastasis and Δα-HBDH after two cycles remained significant predictors of 1-year progression-free survival (both *P* < 0.05). Patients were stratified into high- and low-level groups on the basis of their baseline LDH and α-HBDH values, with the upper limit of the institutional reference range at Shandong Cancer Hospital (LDH: 245 U/L; α-HBDH: 182 U/L) used as the cutoff threshold. Changes (from baseline to 2 cycles) were categorized on the basis of the optimal cutoff values for the entire cohort. Kaplan–Meier curves with log-rank tests were employed to assess differences in OS and PFS between all predefined LDH/α-HBDH subgroups (*p* < 0.05). Elevated baseline serum LDH (LDH >245 U/L) and α-HBDH (α-HBDH >182 U/L) were significantly associated with poor OS (p = 0.046, HR = 1.798, 95% CI: 1.020–3.167; p = 0.007, HR = 2.268, 95% CI: 1.288–3.994; **Figure 2**). Inadequate reductions in the two-cycle changes in LDH (ΔLDH ≤ 108.5 U/L) and α-HBDH (Δα-HBDH ≤ 62.5 U/L) were associated with poorer OS (p<0.001, HR = 2.561; 95% CI: 1.291–5.080; p<0.001, HR = 2.807; 95% CI: 1.457–5.411; **Figure 3**). For progression-free survival, no significant difference was observed between patients with baseline serum LDH >245 U/L or α-HBDH >182 U/L and those with levels below these cutoffs. Similarly, an on-treatment increase (ΔLDH>12.5 U/L or Δα-HBDH>0.5 U/L) was not predictive compared to a smaller increase or decrease. (P = 0.768, HR = 1.055, 95% CI: 0.739–1.507 for baseline LDH; P = 0.529, HR = 1.121, 95% CI: 0.785–1.601 for baseline α-HBDH; P = 0.115, HR = 0.719, 95% CI: 0.457–1.131 for ΔLDH; P = 0.094, HR = 0.730, 95% CI: 0.494–1.080 for Δα-HBDH; **Figures 4**, **5**).

**Table 2:** Results of univariate and multivariate analyses of factors influencing the overall survival of all the patients.

Variable	Univariate analysis			Multivariate analysis		
	*P*	HR	95% CI	*P*	HR	95% CI
Sex						
Male	.001	0.155	0.038-0.638	.016	0.175	0.042-0.725
Female						
Age						
≥65	.016	2.000	1.135-3.524	.023	1.948	1.095-3.465
<65						
Smoking status						
Never	.55	1.195	0.666-2.144			
Former or Current						
T stage						
4	.961	1.014	0.571-1.801			
1-3						
N stage						
3	.184	1.472	0.832-2.604			
0-2						
Liver metastasis						
No	.082	1.658	0.937-2.933			
Yes						
Bone metastasis						
No	.159	1.551	0.842-2.856			
Yes						
Brain metastasis						
No	.738	0.897	0.475-1.696			
Yes						
Adrenal gland metastasis						
No	.247	0.578	0.229-1.461			
Yes						
LDH before treatment						
>245	.059	1.757	0.980-3.153			
≤245						
α-HBDH before treatment						
>182	.009	2.269	1.232-4.178	.748	1.139	0.514-2.525
≤182						
ΔLDH in 2 cycles						
>108.5	.001	2.563	1.443-4.551			
≤108.5						
Δα-HBDH in 2 cycles						
>62.5	.001	2.812	1.595-4.957	.049	2.046	0.918-4.559
≤62.5						

LDH, lactate dehydrogenase; α-HBDH, α-hydroxybutyrate dehydrogenase; CI, confidence interval; HR, hazard ratio

**Table 3:** Results of univariate and multivariate analyses of factors influencing the progression-free survival of all the patients.

Variable	Univariate analysis			Multivariate analysis		
	*P*	HR	95% CI	*P*	HR	95% CI
Sex						
Male	.300	0.78	0.487-1.249			
Female						
Age						
≥65	.324	1.206	0.831-1.751			
<65						
Smoking status						
Never	.372	1.181	0.819-1.703			
Former or Current						
T stage						
4	.112	1.338	0.934-1.916			
0-3						
N stage						
3	.855	1.034	0.724-1.477			
0-2						
Liver metastasis						
No	.175	1.294	0.891-1.879			
Yes						
Bone metastasis						
No	<0.01	2.019	1.368-2.982	<0.01	2.098	1.400-3.145
Yes						
Brain metastasis						
No	.614	1.105	0.750-1.626			
Yes						
Adrenal gland metastasis						
No	.859	1.046	0.641-1.706			
Yes						
LDH before treatment						
>245	.805	1.046	0.732-1.494			
≤245						
α-HBDH before treatment						
>182	.530	1.121	0.785-1.603			
≤182						
ΔLDH in 2 cycles						
>12.5	.117	0.718	0.475-1.086			
≤12.5						
Δα-HBDH in 2 cycles						
>0.5	.096	0.730	0.504-1.058	.050	0.632	0.399-1.002
≤0.5						

LDH, lactate dehydrogenase; α-HBDH, α-hydroxybutyrate dehydrogenase; CI, confidence interval; HR, hazard ratio

### 3.3 Clinical correlates of LDH and α-HBDH

Mann–Whitney U tests revealed significant associations between subgroup stratification and longitudinal LDH/α-HBDH dynamics. As shown in **Table 4**, in terms of T stage (T1–3 vs. T4), differential 2-cycle ΔLDH/Δα-HBDH trajectories were observed, with higher ΔLDH and Δα-HBDH values at 2 cycles in the T1–3 group than in the T4 group (p=0.007 for ΔLDH, p=0.011 for Δα-HBDH). Compared with N0–2 patients, N3 patients exhibited elevated baseline LDH/α-HBDH (p=0.010 for LDH, p=0.037 for α-HBDH) and blunted biomarker reductions at 2 cycles (p=0.001 for ΔLDH, p=0.006 for Δα-HBDH). Patients aged **≥** 65 years had higher pretreatment LDH (median: 235.5 vs. 268 U/L) and α-HBDH (median: 170 vs. 195 U/L) levels than younger individuals did (p=0.020 for LDH and p=0.022 for α-HBDH).

**Table 4:** Association between clinical subgroups and longitudinal LDH/α-HBDH dynamics.

Variable	Level and p	LDH before treatment	ΔLDH in 2 cycles	α-HBDH before treatment	Δα-HBDH in 2 cycles
T stage Median/Mean (P25, P75)	1-3(n=119)	238.0(206.5,301.5)	20.0(-15.5,84.5)	175.0(150.0,218.5)	14.0(-13.5,59.5)
	4(n=82)	257.5(214.0,350.5)	50.5(11.0,126.8)	195.0(158.0,236.4)	35.5(4.0,80.0)
	*p*	0.13	0.007*	0.173	0.011*
N stage Median/Mean (P25, P75)	0-2(*n* = 105)	231.0 (200.5,283.5)	17.0(-18.0,68.0)	176.0(143.0,211.5)	13.0(-18.0,53.5)
	3(*n* = 96)	269.5(219.0,355.8)	63.5(3.5,142.0)	191.5(162.3,241.3)	36.0(-0.5,87.3)
	*p*	0.010*	0.001*	0.037*	0.006*
Age Median/Mean (P25, P75)	≥65(*n* = 134)	235.5(199.3,305.8)	28.0(-10.5,107.8)	170.0(143.8,221.0)	18.0(-11.3,68.3)
	<65 (*n* = 67)	268.0(220.0,342.0)	47.0(5.0,109.0)	195.0(167.0,237.0)	28.0(3.0,64.0)
	*p*	0.020*	0.172	0.022*	0.266
Immune-Mediated Myocarditis Median/Mean (P25, P75)	No(n=173)	245.0(208.0,320.0)	35.0(-7.0,107.0)	183.0(153.0,228.0)	22.0(-6.0,68.0)
	Yes(*n* = 28)	244.0(198.5,305.3)	27.5(-5.8,75.5)	169.0(140.5,207.3)	13.0(-12.0,58.3)
	*p*	0.735	0.86	0.658	0.437
Immune-Mediated Pneumonitis Median/Mean (P25, P75)	No(*n* = 173)	251.0(208.0,324.0)	38.0(-8.0,111.5)	186.0(154.0,233.5)	27.0(-7.5,68.0)
	Yes(*n* = 28)	244.0(198.5,305.3)	27.5(-5.8,75.5)	169.0(140.5,207.3)	13.0(-12.0,58.3)
	*p*	0.483	0.63	0.284	0.378
Immune-Mediated Hypothyroidism Median/Mean (P25, P75)	No(n=166)	247.5(208.0,324.0)	28.0(-10.0,114.5)	186.0(156.5,231.3)	20.5(-9.0,68.0)
	Yes(n=35)	249.0(201.0,295.0)	50.0(17.0,78.0)	170.0(142.0,228.0)	26.0(7.0,64.0)
	*p*	0.642	0.303	0.282	0.576
Immunotherapy drugs Median/Mean (P25, P75)	No(n=116)	243.5(207.0,332.3)	38.5(-7.0,125.5)	177.0(150.3,231.3)	22.0(-6.0,74.3)
	Yes(n=85)	253.0(207.5,317.5)	33.0(-9.0,93.0)	188.0(158.0,230.5)	22.0(-11.5,64.5)
	*p*	0.833	0.52	0.724	0.749
pretreatment pneumonia Median/Mean (P25, P75)	No(n=123)	244.0(206.0,335.0)	33.0(-9.0,124.0)	178.0(150.0,234.0)	20.0(-8.0,83.0)
	Yes(n=78)	250.500(208.0,309.5)	38.500(-5.5,100.8)	187.5(155.3,222.3)	33.0(-6.5,63.3)
	*p*	0.867	0.845	0.871	0.661

LDH, lactate dehydrogenase; α-HBDH, α-hydroxybutyrate dehydrogenase;

*p < 0.05.

No significant variations in LDH or α−HBDH levels were observed in association with immune−mediated adverse events (including myocarditis, pneumonitis, or thyroid dysfunction), different immunotherapy regimens, or history of pretreatment pneumonia (all p > 0.05). Binary logistic regression confirmed that nodal metastasis and advanced age were independent predictors of aberrant LDH/α−HBDH profiles, which aligns with the findings from the univariate analysis (**Table 5**).

**Table 5:** Results of the bivariate regression analysis of LDH and α-HBDH levels in relation to clinical subgroups.

Variable	LDH before treatment	ΔLDH in 2 cycles	α-HBDH before treatment	Δα-HBDH in 2 cycles
Immune-Mediated Pneumonitis	0.698	0.495	0.401	0.835
Immune-Mediated Myocarditis	0.590	0.487	0.575	0.660
Immune-Mediated Hypothyroidism	0.939	0.131	0.451	0.921
T stage	0.601	0.079	0.051	0.355
N stage	0.050*	0.002*	0.115	0.138
Immunotherapy Drugs	0.660	0.280	0.524	0.505
Age	0.088	0.847	0.049*	0.792
Pretreatment Pneumonia	0.518	0.638	0.267	0.139

LDH, lactate dehydrogenase; α-HBDH, α-hydroxybutyrate dehydrogenase

*p < 0.05.

## 4 Discussion

ES-SCLC represents a global health crisis because of its dismal clinical outcomes and historically stagnant therapeutic innovation. Despite the establishment of chemoimmunotherapy as the first-line standard of care, five-year survival rates remain unacceptably low (4). Thus, numerous investigations are underway to improve therapeutic efficacy. For instance, the ETER701 study aimed to evaluate whether the addition of benmelstobart and anlotinib to standard chemotherapy confers synergistic benefits in the first-line treatment of ES-SCLC. Promisingly, this regimen is associated with a median OS of 19.3 months and a median PFS of 6.9 months, representing unprecedented improvements in both endpoints (18). Consequently, the early prediction of immunotherapy response and timely intervention are critical. In cancer patients, routine radiological examinations (e.g., CT scans) remain the standard for assessing treatment response for most cancer types. Early identification of disease progression remains challenging. This is partly due to the RECIST criteria, which define progression as a ≥20% increase in tumor size—a threshold that is relatively insensitive. This diagnostic gap underscores the importance of identifying LDH and α-HBDH levels, which reflect real-time tumor biological activity. In this study, serum LDH and α-HBDH levels were identified as key prognostic biomarkers in ES-SCLC patients receiving first-line chemoimmunotherapy. Clinically validated thresholds were defined by elevated absolute levels of LDH and α-HBDH that failed to decrease sufficiently after two treatment cycles. This profile strongly predicted poor survival outcomes. These findings underscore the importance of these biomarkers in guiding subsequent treatment strategies with greater precision.

LDH and its isoenzyme α-HBDH maintain the Warburg effect by catalyzing the reduction of pyruvate to lactate with concomitant NAD^+^ regeneration, thereby sustaining glycolytic flux in tumor cells. Their synergistic upregulation enables tumors to adapt to nutritional deficiency, thereby promoting rapid proliferation and therapeutic evasion. Elevated levels of LDH and α-HBDH indicate enhanced hypoxia-driven metabolism in tumors. The lactate overproduction induced by this process acidifies TME, suppresses cytotoxic T-cell activity, and promotes macrophage polarization toward protumor phenotypes via upregulation of pH-sensitive PD-L1 (12–14). Tumor-derived elevations in LDH and α-HBDH promote enhanced glycolytic flux, metastatic competence, and immune evasion in preclinical SCLC models (19). Our findings reinforce serum LDH as a fundamental prognostic biomarker in immuno-oncology, consistent with its role in other malignancies such as melanoma treated with checkpoint inhibitors (20, 21). Our study not only confirms this finding in ES-SCLC but also reveals two enhanced clinical applications. First, it demonstrates the specific prognostic value of the α-HBDH isoenzyme. Second, it shows that early kinetic changes—specifically, an insufficient decrease after two treatment cycles—provide dynamic prognostic information. This approach aligns with the clinical rationale of using readily available biomarkers—like the neutrophil-to-lymphocyte ratio (NLR) in melanoma—for risk stratification (21). While NLR reflects systemic inflammation, LDH and α-HBDH directly report tumor glycolytic activity and microenvironmental stress. Together, these accessible metrics could provide a composite, real-time profile of tumor biology. Future validation of a combined model integrating metabolic (LDH/α-HBDH kinetics) and inflammatory (e.g., NLR) markers could offer a pragmatic tool for personalizing therapy in ES-SCLC.

Therefore, we chose them for our study. We aimed to systematically evaluate the prognostic utility of longitudinal LDH/α-HBDH dynamics in ES-SCLC patients on first-line chemoimmunotherapy. Based on our findings, we established an evidence-based framework for adaptive risk stratification and therapeutic monitoring. Our findings reveal that LDH and α-HBDH are pivotal prognostic indicators for patients with ES-SCLC. The strong correlation between elevated baseline levels (LDH >245 U/L, α-HBDH >182 U/L) and reduced OS further supports the hypothesis that acidosis in the TME facilitates immune evasion, thereby contributing to poor clinical outcomes. Our study pioneers the integration of longitudinal LDH and α-HBDH dynamics into ES-SCLC prognosis, addressing a critical gap in existing static biomarker paradigms. In contrast to previous investigations restricted to baseline assessments, serial monitoring at 2-cycle intervals revealed time-dependent metabolic signatures with enhanced prognostic discrimination. Inadequate reductions in the two-cycle changes in LDH (ΔLDH ≤ 108.5 U/L) and α-HBDH (Δα-HBDH ≤ 62.5 U/L) predicted poorer OS. The lack of significant associations between LDH/α-HBDH profiles and PFS may be attributed to the 1-year follow-up window, which was insufficient to capture long-term progression events. These clinical observations are consistent with mechanistic studies that have demonstrated LDH/α-HBDH-mediated modulation of the TME. The LDH-A isoform reprograms the TME by driving lactate overproduction. This lactate buildup promotes the expansion of immune-suppressive cells (e.g., myeloid-derived suppressor cells, tumor-associated macrophages, and dendritic cells) while inhibiting cytolytic cells, such as natural killer cells and cytotoxic T lymphocytes. This LDH-A-driven immunosuppressive milieu potentiates resistance to diverse anticancer therapies (22, 23).

This temporal stratification establishes clinically actionable thresholds to guide adaptive therapeutic intensification. Attainment of these absolute thresholds or failure to achieve specified reduction magnitudes enabled earlier identification of high-risk patients. Based on these mechanistic insights, we propose a clinically actionable dynamic monitoring framework for LDH/α-HBDH. If post-treatment levels remain above validated thresholds or fail to achieve predefined reductions, acidosis-targeting interventions should be considered. Such strategies may include low-dose radiotherapy to reduce tumor burden and modulate the immune microenvironment, combined with anti-angiogenic agents to counteract lactate accumulation. The aim of this combined approach is to ultimately remodel the immunosuppressive landscape, thereby potentiating immunotherapy efficacy and improving survival outcomes in patients with chemoimmunotherapy-resistant ES-SCLC (24).

Subgroup analyses revealed significant associations between tumor burden (T stage), nodal involvement (N stage), age≥65 years and LDH/α-HBDH levels. When stratified by tumor burden, T4 tumors showed a weaker reduction in LDH/α-HBDH compared to T1–3 tumors. This aligns with the established link between LDH levels and metabolic tumor volume (25). The likely reason is that larger tumor volumes intensify Warburg effect-driven acidosis. The resulting increase in microenvironment acidification may, in turn, impair the efficacy of immunotherapy. Baseline LDH and α-HBDH levels were elevated in patients aged ≥ 65 years, suggesting that elderly individuals may experience lower treatment efficacy because of these heightened baseline values. Notably, a comparative analysis of nodal metastasis stratification between N3 and N0–2 patients revealed significantly higher absolute baseline levels of LDH and α-HBDH and attenuated reductions in the magnitude of change at the 2-cycle timepoint. This pattern likely reflects progressively intensified tumor microenvironment acidosis, in which greater acidification potentiates metastatic progression (26). Our findings are consistent with previous evidence that high LDH and α-HBDH levels are positively associated with lymph node metastasis in a variety of tumors (27).

LDH and α-HBDH levels showed no association with immune-related adverse events. This is likely because these enzymes mainly reflect the tumor’s own glycolytic activity and the body’s overall metabolic burden. Immune toxicity, in contrast, comes from off-target T-cell attacks on healthy tissues. These are fundamentally different biological processes. In contrast, nodal metastasis and advanced age emerged as independent predictors of elevated LDH and α-HBDH, consistent with their roles in driving increased tumor burden and chronic, low-grade metabolic inflammation, respectively (28).

This study has several limitations. First, the single-center retrospective design leads to selection bias and unmeasured confounding variables from regional practice variations; validation in multicenter cohorts with diverse populations is needed to enhance the generalizability of the study findings. Second, while univariate analysis identified prognostic factors for 1-year OS and PFS, these factors were not significant in the multivariate Cox model. This may be because serum LDH/HBDH levels are influenced by many non-cancer conditions. This multifactorial nature likely diluted their statistical power as independent predictors. Third, the current 1-year follow-up period limits the assessment of long-term outcomes, and the potential biological variability of LDH and α-HBDH — stemming from individual metabolic differences or concurrent non-malignant conditions — may affect the reproducibility and broader clinical applicability of the findings. Finally, although patients with disorders that are known to increase LDH/α-HBDH levels (e.g., hemolysis and myocardial infarction) were excluded, undiagnosed comorbidities or subclinical inflammatory states may compromise the specificity of these biomarkers for tumor metabolism.

## 5 Conclusion

This study establishes serum LDH and α-HBDH levels as pivotal prognostic biomarkers in ES−SCLC patients receiving first−line chemoimmunotherapy. We defined clinically applicable thresholds, demonstrating that elevated baseline levels and inadequate reduction after 2 treatment cycles consistently predict poor survival. Building on these mechanistic insights, we propose that dynamic monitoring of LDH/α-HBDH may help identify patients who could benefit from adjunctive interventions. Interventions such as low-dose radiotherapy or anti-angiogenic agents aim to target tumor-associated acidosis and remodel the immunosuppressive microenvironment. Future multicenter prospective trials are warranted to validate these thresholds and assess the clinical utility of this integrated biomarker−guided approach.

## Data Availability

The raw data supporting the conclusions of this article will be made available by the authors, without undue reservation.
